# Intravoxel Incoherent Motion Diffusion-Weighted Imaging Used to Detect Prostate Cancer and Stratify Tumor Grade: A Meta-Analysis

**DOI:** 10.3389/fonc.2020.01623

**Published:** 2020-09-11

**Authors:** Ni He, Zhipeng Li, Xie Li, Wei Dai, Chuan Peng, Yaopan Wu, Haitao Huang, Jianye Liang

**Affiliations:** ^1^Department of Medical Imaging, Sun Yat-sen University Cancer Center, State Key Laboratory of Oncology in South China, Collaborative Innovation Center for Cancer Medicine, Guangzhou, China; ^2^Department of Radiology, Maoming People's Hospital, Maoming, China; ^3^Medical Imaging Center, The First Affiliated Hospital of Jinan University, Guangzhou, China

**Keywords:** intravoxel incoherent motion diffusion-weighted imaging, post-test probability, diagnostic performance, prostate cancer, gleason grade, meta-analysis

## Abstract

**Objectives:** Intravoxel incoherent motion diffusion-weighted imaging (IVIM-DWI) is a promising non-invasive imaging technique to detect and grade prostate cancer (PCa). However, the results regarding the diagnostic performance of IVIM-DWI in the characterization and classification of PCa have been inconsistent among published studies. This meta-analysis was performed to summarize the diagnostic performance of IVIM-DWI in the differential diagnosis of PCa from non-cancerous tissues and to stratify the tumor Gleason grades in PCa.

**Materials and Methods:** Studies concerning the differential diagnosis of prostate lesions using IVIM-DWI were systemically searched in PubMed, Embase, and Web of Science without time limitation. Review Manager 5.3 was used to calculate the standardized mean difference (SMD) and 95% confidence intervals of the apparent diffusion coefficient (ADC), tissue diffusivity (D), pseudodiffusivity (D^*^), and perfusion fraction (f). Stata 12.0 was used to pool the sensitivity, specificity, and area under the curve (AUC), as well as publication bias and heterogeneity. Fagan's nomogram was used to predict the post-test probabilities.

**Results:** Twenty studies with 854 patients confirmed with PCa were included. Most of the included studies showed a low to unclear risk of bias and low concerns regarding applicability. PCa showed a significantly lower ADC (SMD = −2.34; *P* < 0.001) and D values (SMD = −1.86; *P* < 0.001) and a higher D^*^ value (SMD = 0.29; *P* = 0.01) than non-cancerous tissues, but no difference was noted with the f value (SMD = −0.16; *P* = 0.50). Low-grade PCa showed higher ADC (SMD = 0.63; *P* < 0.001) and D values (SMD = 0.80; *P* < 0.001) than the high-grade lesions. ADC showed comparable diagnostic performance (sensitivity = 86%; specificity = 86%; AUC = 0.87) but higher post-test probabilities (60, 53, 36, and 36% for ADC, D, D^*^, and f values, respectively) compared with the D (sensitivity = 82%; specificity = 82%; AUC = 0.85), D^*^ (sensitivity = 70%; specificity = 70%; AUC = 0.75), and f values (sensitivity = 73%; specificity = 68%; AUC = 0.76).

**Conclusion:** IVIM parameters are adequate to differentiate PCa from non-cancerous tissues with good diagnostic performance but are not superior to the ADC value. Diffusion coefficients can further stratify the tumor Gleason grades in PCa.

## Introduction

Prostate cancer (PCa) remains the most frequently diagnosed cancer and is the second leading cause of cancer death among men in the United States in 2020 (1). Early diagnosis of PCa and stratification of tumor grades are important for risk assessment and management strategies. PCa patients with high Gleason scores usually accept radical prostatectomy and radiation therapy, whereas patients with low-risk cancer are optimal for active surveillance instead of immediate intervention, particularly in older men (2). A previous meta-analysis reported that multiparametric magnetic resonance imaging (MRI) improved the accuracy of PCa detection and local staging (3). The diffusion-weighted imaging (DWI)-derived apparent diffusion coefficient (ADC) has become a valuable quantitative parameter to detect and grade PCa (4). However, the ADC value may overlap between PCa and non-cancerous tissues because benign prostatic hyperplasia also shows increased cellularity, and ADC is mixed with microcirculation perfusion within the capillaries.

Intravoxel incoherent motion (IVIM), which was first introduced by Le Bihan et al. (5), can separate the incoherent motion of water molecules within the capillaries from extravascular molecular diffusion. The true diffusion coefficient (D value), pseudodiffusion coefficient (D^*^ value), and perfusion fraction (f value) are generated using a biexponential model with multiple *b*-values. Previous studies have indicated that IVIM parameters have potential values in the diagnosis of PCa and show a close relationship with the Gleason score and tumor aggressiveness (6, 7). However, the diagnostic performance of IVIM-DWI-derived parameters in the detection of PCa is not consistent, and its application remains contentious. For example, most studies (8–10) have indicated that PCa has a higher D^*^ value than non-cancerous tissues, whereas some studies have reported adverse (11–13) or insignificant results (14, 15). Numerous studies have reported a significantly lower f value in PCa than in non-cancerous tissues (10, 14, 16, 17). Furthermore, some studies have indicated that the ADC value provides better diagnostic performance than IVIM parameters for PCa detection or grading (2, 4, 6, 16). These contentious results may derive from the small sample sizes in individual studies. To address this problem, we performed a meta-analysis to pool all the published results concerning the diagnostic performance of IVIM-DWI in the detection of PCa from non-cancerous tissues and to stratify the tumor Gleason grades in PCa. Thus, the controversial issues among different studies will be addressed with more reliable evidence.

## Materials and Methods

### Data Sources

Two senior librarians systemically retrieved studies on the detection and stratification of PCa using IVIM-DWI parameters from PubMed, Embase, and Web of Science without time limitation. A searching formula was formed using different combinations of medical subject headings or keywords from IVIM, multiple b-value DWI, biexponential, and prostate or PCa/carcinoma/tumor. The primary searches were limited in the titles and abstracts. We also performed manual retrieval of the reference lists from the included studies.

### Study Selection

Studies meeting the following criteria were included: (a) IVIM-DWI parameters were used to differentiate PCa from non-cancerous tissues or low-grade from high-grade PCa; (b) the mean and standard deviation (SD) of each parameter was provided; (c) the diagnostic performance concerning sensitivity and specificity was reported; (d) PCa was confirmed by pathology after initial MRI examination but before treatment; and (e) at least one b-value <200 mm^2^/s, and all the *b*-values were not larger than 3,000 mm^2^/s. The exclusion criteria were as follows: (a) duplication from the same authors or institutions; (b) meta-analyses, conference abstracts, reviews, or any unpublished results; and (c) animal experiments or non-prostate studies and (d) non-English studies.

### Data Extraction

A spreadsheet was used to extract the mean values and SD, as well as the diagnostic performance of ADC, D, D^*^, and f values with a threshold value, area under the curve (AUC), sensitivity, and specificity in each study by one author and then, was reviewed by another. Other information included the first author, publication years, countries, field strength, and vendors, *b*-values, repetition times, echo times, acceleration factor, patient ages and numbers, and prostate-specific antigen level. True-positive, false-negative, false-positive, and true-negative data were calculated when the number of PCa and non-cancerous tissues, as well as the sensitivity and specificity or receiver operating curve, were provided.

### Quality Assessment

The quality of studies and the likelihood of bias were evaluated using Review Manager 5.3 software (Cochrane Collaboration, Oxford, UK), based on the Quality Assessment of Diagnostic Accuracy Studies-2 (18). We assessed the risk of bias and applicability concerns in four domains, including patient selection, index tests, reference standard, and flow, and timing (19).

### Publication Bias and Heterogeneity Evaluation

Because two parts of the data were pooled in our study—quantitative values and diagnostic performance of each parameter, funnel plots, and Begg's test were used to visually and quantitatively assess the publication bias for the continuous variables, and Deeks' plot assessed the publication bias regarding diagnostic performance using Stata version 12.0 (StataCorp LP, College Station, TX). For an asymmetric or skewed funnel plot, *P* < 0.05 in Begg's test or Deeks' test indicated the potential of publication bias (20). The inconsistency index (*I*^2^) and Cochran's *Q*-tests were used to explore the heterogeneity of the included studies, with *I*^2^ > 50% or *P* < 0.05 for Cochran' *Q*-test, suggesting statistically significant heterogeneity and a random-effects model was applied in subsequent pooling, or a fixed-effects model when *I*^2^ <50% (21).

### Data Synthesis

We constructed forest plots for continuous variables and calculated the standardized mean difference (SMD) between PCa and non-cancerous tissues using Review Manager 5.3 software. We used the bivariate regression model to pool the diagnostic performance with sensitivity, specificity, positive likelihood ratio (PLR), negative likelihood ratio (NLR), diagnostic odds ratio (DOR), and AUC using Stata version 12.0. The likelihood ratio and post-test probability are also important to diagnose disease (22), providing the likelihood that a patient was diagnosed with a certain disease using MRI parameters. Summary receiver operating characteristic curves and Fagan's nomograms were also plotted to determine the diagnostic values and predict the post-test probabilities of ADC, D, D^*^, and f values in the detection of PCa.

## Results

### Literature Search and Selection

**Figure 1:**
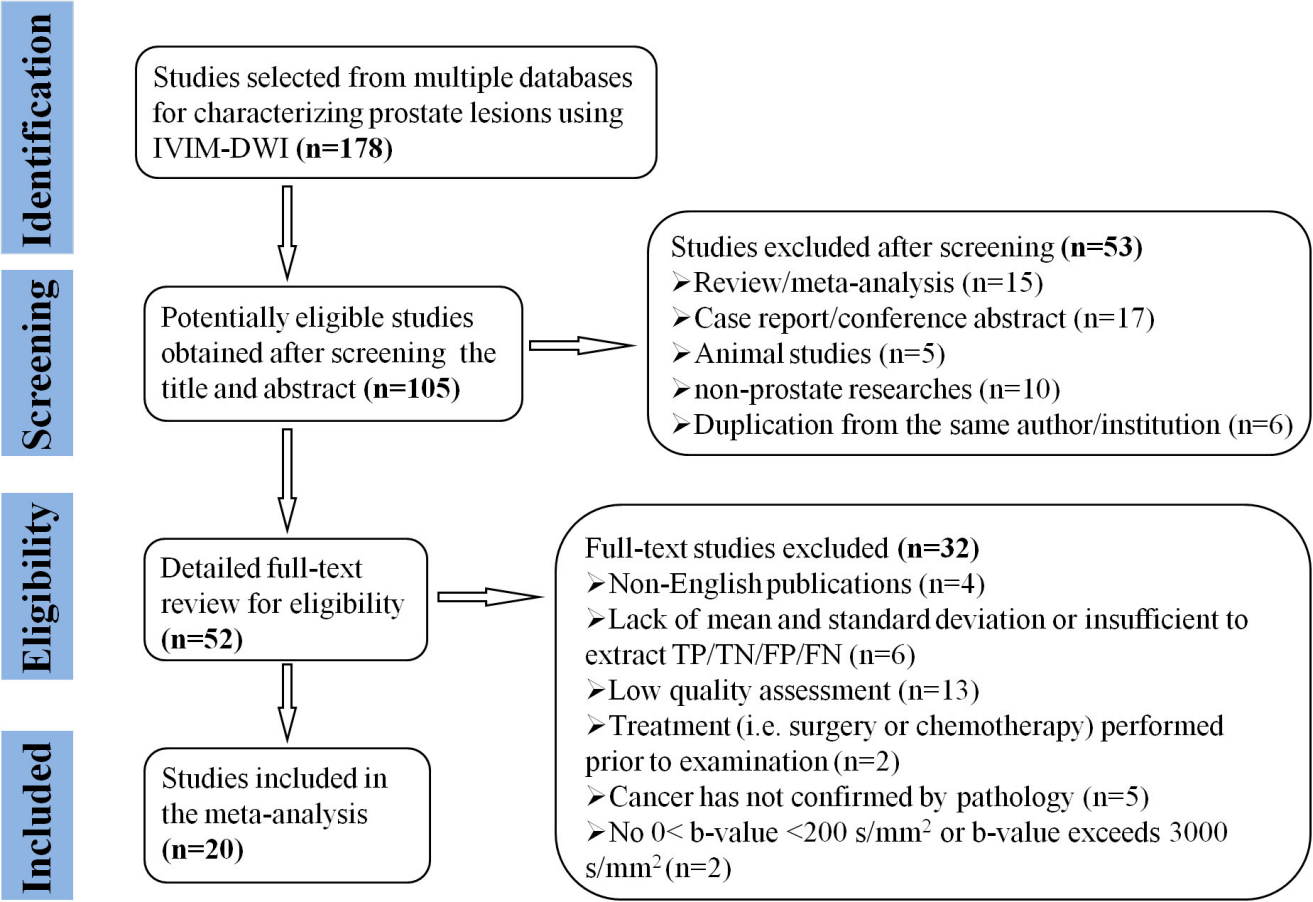
Flowchart detailing the study selection process. Twenty studies that met the inclusion criteria were included. FN, false negative; FP, false positive; TN, true negative; TP, true positive.

Searching the keywords in the titles and abstracts returned 178 potential studies from multiple databases. Thirty-two studies regarding meta-analyses, conference abstracts, case reports, and reviews were excluded after screening the titles and abstracts. Animal studies, non-prostate studies, and duplication from the same authors or institutions led to further exclusion of 21 studies. We scrutinized the full-texts of the remaining 52 studies in detail and excluded an additional 32 studies for the following reasons: (a) non-English studies; (b) lack of sufficient data to be pooled; (c) low-quality assessment; (d) IVIM-DWI was interfered by treatment; (e) cancer was not confirmed by pathology; and (f) no 0 < *b*-value < 200 s/mm^2^ or *b*-value exceeds 3,000 s/mm^2^. Eventually, 20 eligible studies with 854 patients confirmed with PCa were included for analysis. The flowchart detailing the process of study selection is provided in Figure 1. The basic information and diagnostic performance for each included study are detailed in Tables 1, 2. All the studies used a single-shot echo-planar imaging pulse sequence and three orthogonal diffusion directions for IVIM-DWI acquisition.

**Table 1:** Basic information for each included study.

**Authors**	**Year**	**Country**	**Machine type**	***b*-values (s/mm^**2**^)**	**TR (ms)**	**TE (ms)**	**AF**	**Patients**	**Age (years)**	**PSA level (ng/ml)**
Bao et al. (23)	2017	China	3-T Siemens	0, 50, 100, 150, 200, 500, 1,000	6,800	98	2	30	Benign: 63 ± 1.5; Malignant: 69 ± 1.5	Benign: 12.90 ± 1.13; Malignant: 72.13 ± 26.93
Beyhan et al. (11)	2019	Turkey	3-T Siemens	0, 50, 100, 150, 200, 300, 400, 500, 600, 700, 800, 900, 1,000, 1,100, 1,200, 1,300	5,600	66	NA	29	65.37 (50–76)	14.85 ± 17.29
Cui et al. (14)	2019	China	3-T GE	0, 20, 50, 100, 200, 500, 1,000, 1,500, 2,000	5,000	60	2	30	Benign: 70.4 ± 9.5; Malignant: 72.3 ± 9.7	Benign: 7.2 ± 2.3; Malignant: 14.7 ± 15.8
Döpfert et al. (16)	2011	Germany	3-T Siemens	0, 50, 500, 800	2,600	66	NA	13	67 (59–75)	10.4 (2.8–21.5)
Kuru et al. (4)	2014	Germany	3-T Siemens	0, 50, 100, 150, 200, 250, 800	3,100	52	2	27	Benign: 62.9 ± 5.8; Malignant: 68.9 ± 6.3	Benign: 7.7 ± 3.1; Malignant: 8.8 ± 4.6
Merisaari et al. (12)	2016	Finland	3-T Philips	0,2, 4, 6, 9, 12, 14, 18, 23, 28, 50, 100, 300, 500	1,394	44	2	81	64 (49–74)	9.3 (1.3–55)
Pang et al. (24)	2012	USA	3-T Philips	0, 188, 375, 563, 750	4,584	59	2	33	61.6 (53–81)	10 (1.32–45)
Pesapane et al. (9)	2017	Italy	1.5-T GE	0, 10, 20, 30, 50, 80, 100, 200, 400, 800	7,000	10	2	31	61.6 (53–78)	10 (4–45)
Riches et al. (10)	2009	UK	1.5-T Philips	0, 1, 2, 4, 10, 20, 50, 100, 200, 400, 800	2,500	69	NA	50	66 ± 6	NA
Shinmoto et al. (17)	2012	Japan	3-T Philips	0, 10, 20, 30, 50, 80, 100, 200, 400, 1,000	5,132	40	2	26	67.3 (60–78)	13.7 (4.1–130.4)
Ueda et al. (13)	2015	Japan	3-T Philips	0, 50, 100, 200, 500, 1,000, 2,000, 3,000	4,000	65	3	63	65.7 ± 6.24	9.03 ± 4.08
Valerio et al. (25)	2016	Italy	3-T GE	0, 10, 20, 30, 40, 50, 80, 100, 200, 400, 800	3,100	102	NA	53	NA	NA
Chen et al. (26)	2020	China	3-T Philips	0, 188, 375, 563, 750	3,000	63	2.5	75	66 (47–89)	11.31 (0.02–424.77)
Yang et al. (6)	2016	Korea	3-T Philips	0, 10, 20, 50, 100, 200, 500, 800	5,000	90	2	41	71 (50–86)	21 (3.9–84.8)
Yuan et al. (15)	2016	USA	3-T Philips	0, 10, 25, 50, 100, 250, 450, 1,000, 1,500, 2,000	7,000	80	NA	43	61 ± 8	12.3 ± 21.9
Zhang et al. (7)	2014	China	3-T Siemens	0, 50, 150, 300, 600, 900	6,000	72	2	48	70 (57–86)	19.2 (0.7–214.4)
Barbieri et al. (2)	2016	Switzerland	3-T Siemens	0, 10, 20, 50, 130, 270, 500, 900	2,600	58	3	89	64 (43–80)	NA
Li et al. (8)	2018	China	3-T GE	0, 20, 40, 80, 100, 150, 200, 400, 800, 1,000, 1,200, 1,500, 2,000	3,000	70	2	27	68.2 ± 6.1	58–81
Mazzoni et al. (27)	2013	Italy	1.5-T Siemens	0, 50, 100, 150, 200, 250, 400, 650, 800, 1,000, 1,400, 1,800, 2,300	2,100	69	3	57	67 (50–83)	9.36 (1.29–32)
Quentin et al. (28)	2012	Germany	3-T Siemens	0, 50, 100, 150, 200, 300, 400, 500, 600, 700, 800	2,600	89	2	8	68.5 (49–74)	NA

*NA, not available; PSA, prostate-specific antigen; TR, repetition time; TE, echo time; AF, acceleration factor*.

**Table 2:** Diagnostic performance for each included study.

**Indicator**	**Authors**	**Year**	**Threshold**	**AUC**	**Sensitivity**	**Specificity**	**TP**	**FP**	**FN**	**TN**
ADC	Bao et al. (23)	2017	NA	NA	0.871	0.8056	26	6	4	27
	Cui et al. (14)	2019	0.851	0.944	0.9375	0.8	15	4	1	16
	Kuru et al. (4)	2014	11.4	0.96	0.852	1	23	0	4	23
	Yang et al. (6)	2016	NA	0.96	0.92	0.854	38	6	3	35
	Li et al. (8)	2018	1.14	0.856	0.704	0.822	19	4	8	18
D	Bao et al. (23)	2017	NA	NA	0.7097	0.7778	21	7	9	26
	Cui et al. (14)	2019	0.436	0.697	0.8125	0.7	13	6	3	14
	Kuru et al. (4)	2014	1.24	0.92	0.815	0.963	22	1	5	22
	Yang et al. (6)	2016	NA	0.956	0.96	0.829	39	7	2	34
	Li et al. (8)	2018	0.51	0.835	0.741	0.822	20	4	7	18
D*	Bao et al. (23)	2017	NA	NA	0.63	0.67	19	11	11	22
	Cui et al. (14)	2019	1.203	0.716	0.75	0.75	12	5	4	15
	Kuru et al. (4)	2014	12.9	0.63	0.482	0.852	13	3	14	20
	Yang et al. (6)	2016	NA	0.725	0.88	0.488	36	21	5	20
f	Bao et al. (23)	2017	NA	NA	0.74	0.68	22	11	8	22
	Cui et al. (14)	2019	48.6	0.881	0.9375	0.75	15	5	1	15
	Kuru et al. (4)	2014	6.6	0.56	0.407	0.852	11	3	16	20
	Yang et al. (6)	2016	NA	0.633	0.84	0.39	34	25	7	16

NA, not available; ADC, apparent diffusion coefficient; D, tissue diffusivity, D^*^, pseudodiffusivity; f, perfusion fraction; AUC, area under the curve; FN, false negative; FP, false positive; TN, true negative; TP, true positive. Threshold values of ADC, D, and D* are factors of 10^−3^ mm^2^/s.

### Quality Assessment

**Figure 2:**
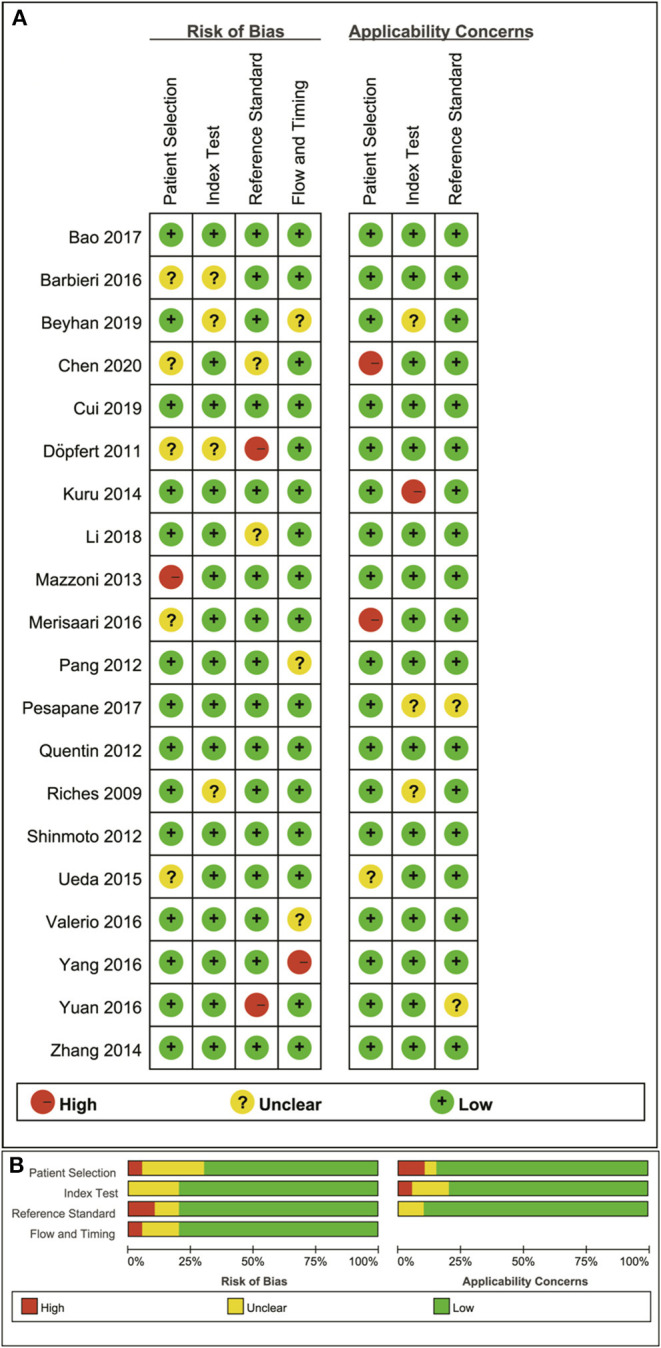
Distribution of risk of bias and applicability concerns for each included study using QUADAS-2 **(A)** and a summary methodological quality **(B)**.

The distribution of Quality Assessment of Diagnostic Accuracy Studie-2 scores for the risk of bias and applicability concerns are shown in Figure 2. The overall quality of the included studies was acceptable. In the patient selection domain, the risk of bias is unclear or high in seven studies because they had not clearly stated whether the patient enrollments were consecutive. The concerns for applicability were considered high because normal tissue in the peripheral zone and benign prostatic hyperplasia were regarded as non-cancerous tissues in three studies. Four studies were marked as unclear risk of bias with high concerns of applicability for the index test domain because the threshold values for ADC, D, D^*^, or f values were not provided. Four studies showed unclear or high risks of bias in the reference standard domain because of unclear biopsy methods selected, such as 12-core systematic transrectal ultrasonography-guided biopsy, MRI, and ultrasonography fusion image-guided biopsy or radical prostatectomy. Six studies demonstrated an unclear risk of bias in the patient flow and timing domain because the intervals between MRI examination and biopsy were not reported.

### Publication Bias and Heterogeneity Analysis

**Figure 3:**
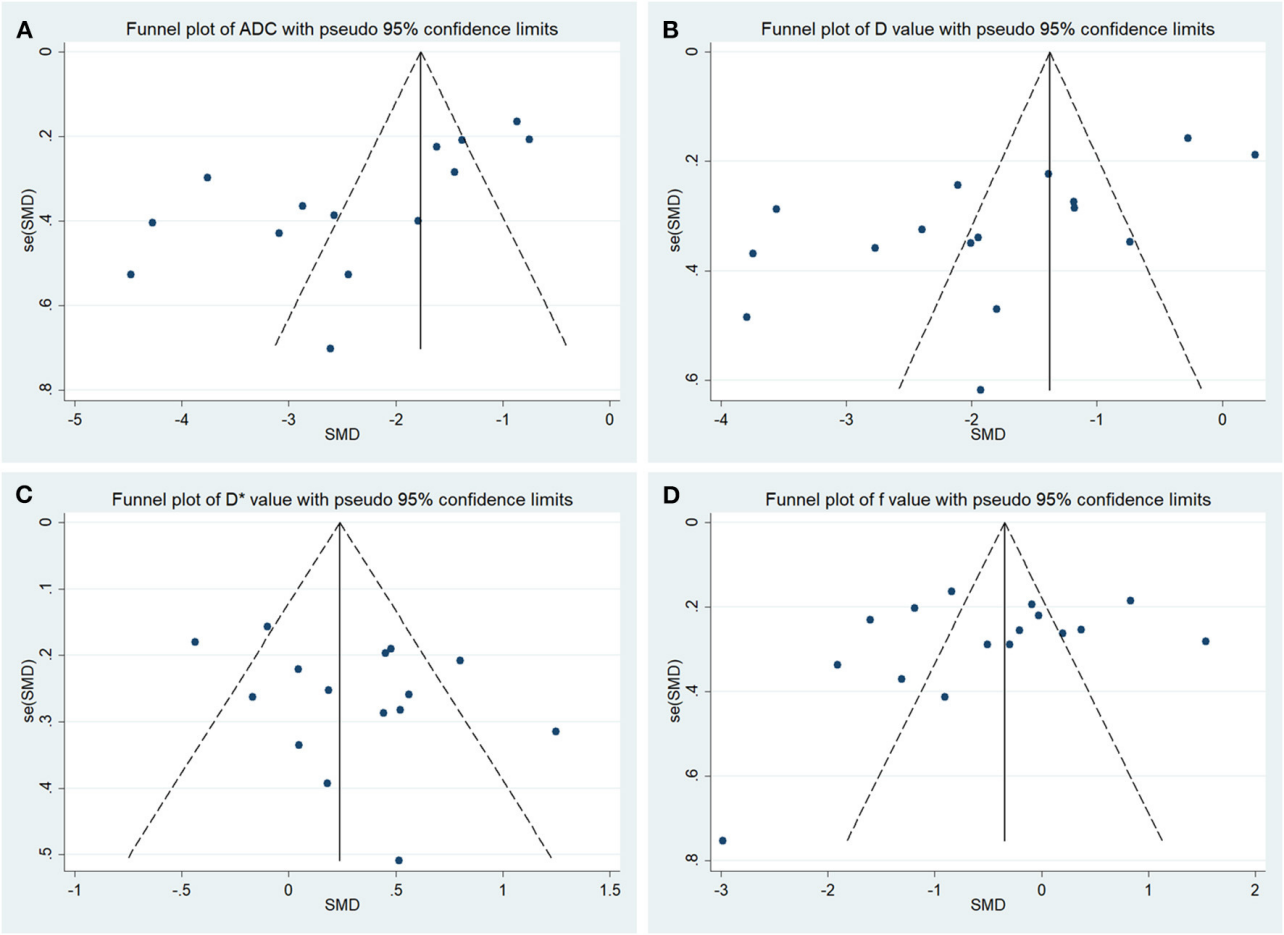
Funnel plots of the **(A)** apparent diffusion coefficient (ADC), **(B)** tissue diffusivity **(D)**, **(C)** pseudodiffusivity (D*), and **(D)** perfusion fraction (f).

The funnel plots depicting the publication bias of ADC, D, D^*^, and f values are shown in Figure 3. An asymmetric funnel plot lacking studies in the right bottom and *P* = 0.022 of Begg's test suggested publication bias in ADC. The funnel plots were basically symmetric with no positive results in Begg's tests (*P* = 0.065, 0.967, and 0.300 for D, D^*^, and f values, respectively), suggesting no obvious publication bias in D, D^*^, and f values.

For the heterogeneity analysis, χ^2^ = 190.72 and *P* < 0.001 of the heterogeneity test with *I*^2^ = 93% suggested high heterogeneity in ADC; χ^2^ = 289.13 and *P* < 0.001 of the heterogeneity test with *I*^2^ = 95% also suggested high heterogeneity in D value; χ^2^ = 44.96 and *P* < 0.001 of the heterogeneity test with *I*^2^ = 69% suggested moderate heterogeneity in D^*^ value; and χ^2^ = 189.44 and *P* < 0.001 of the heterogeneity test with *I*^2^ = 92% suggested high heterogeneity in f value.

## Quantitative Analysis

### Detection of Prostate Lesions Using Apparent Diffusion Coefficient

**Figure 4:**
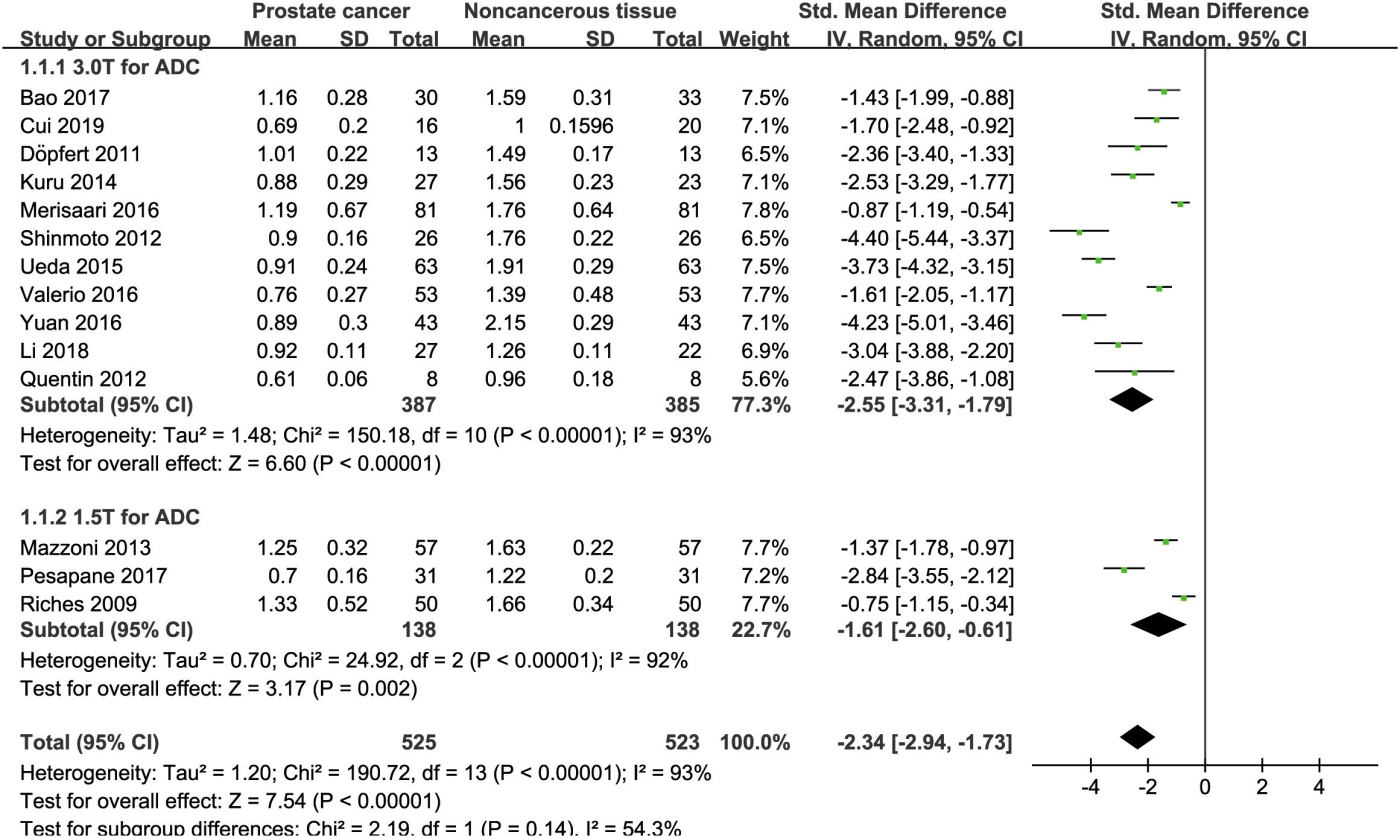
Forest plot of the mean value of the apparent diffusion coefficient (ADC) between prostate cancer and non-cancerous tissues.

Fourteen studies concerning ADC used to detect PCa were included for analysis. The forest plot in Figure 4 shows the distribution of ADC between PCa and non-cancerous tissues. A random-effects model generated an SMD of −2.34 (−2.94, −1.73) (*P* < 0.001) between PCa and non-cancerous tissues for ADC.

### Detection of Prostate Lesions Using the D Value

**Figure 5:**
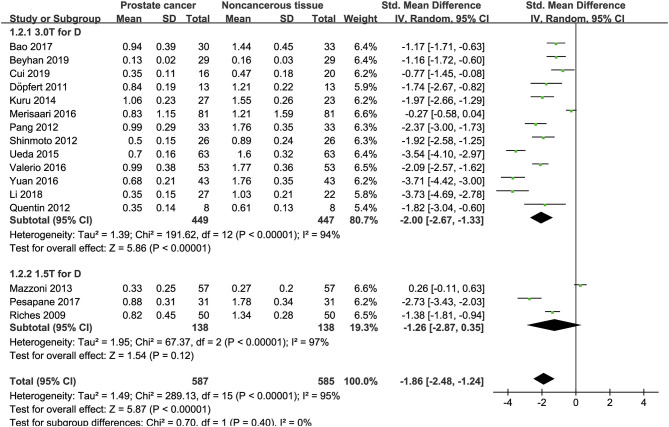
Forest plot of the mean value of the tissue diffusivity (D) between prostate cancer and non-cancerous tissues.

Sixteen studies concerning the D value used to detect PCa were included for analysis. The forest plot in Figure 5 shows the distribution of the D value between PCa and non-cancerous tissues. A random-effects model generated an SMD of −1.86 (−2.48, −1.24) (*P* < 0.001) between PCa and non-cancerous tissues for the D value.

### Detection of Prostate Lesions Using the D^*^ Value

**Figure 6:**
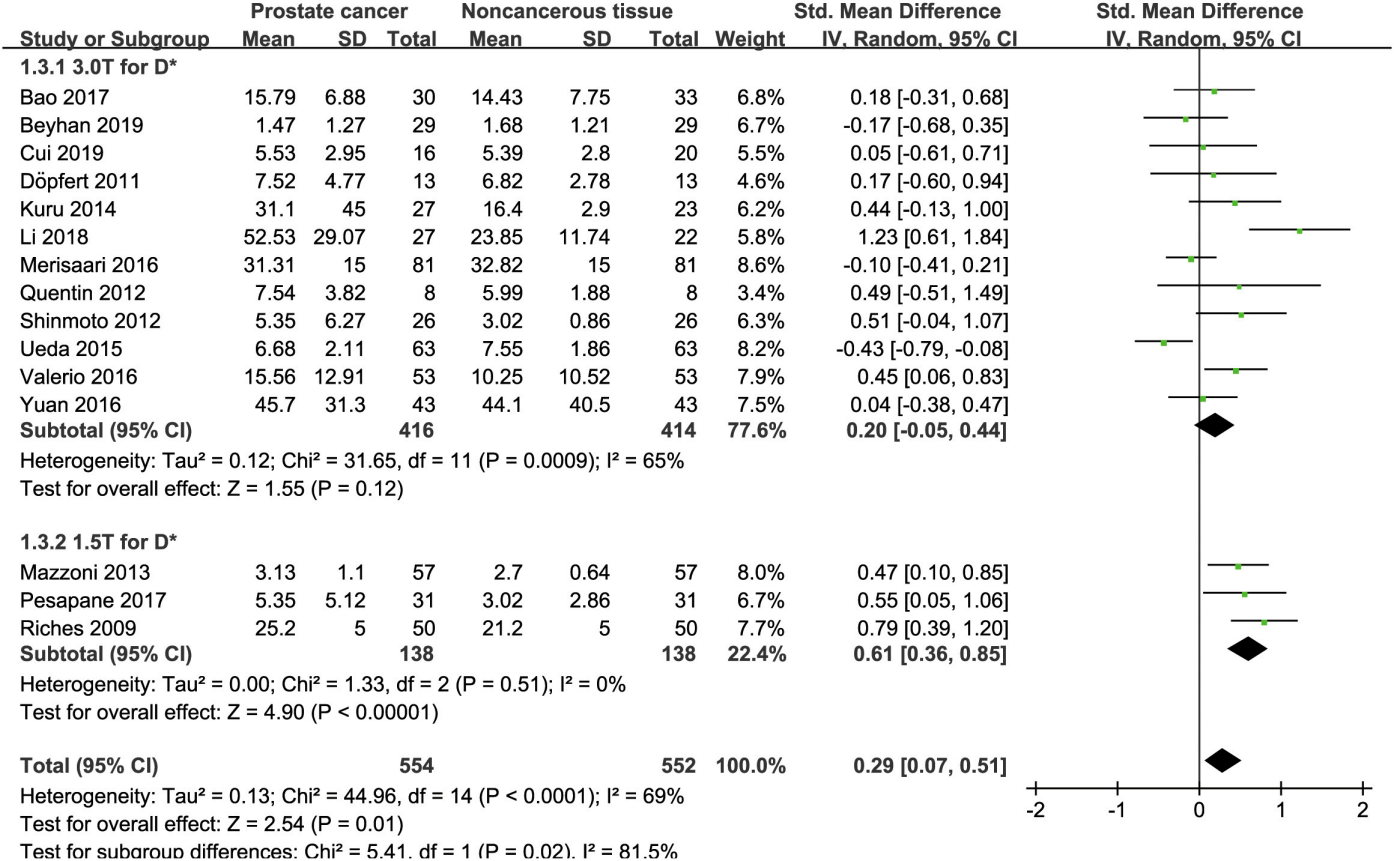
Forest plot of the mean value of the pseudodiffusivity (D*) between prostate cancer and non-cancerous tissues.

Fifteen studies concerning the D^*^ value used to detect PCa were eventually included for analysis. The forest plot in Figure 6 shows the distribution of the D^*^ value between PCa and non-cancerous tissues. A random-effects model generated an SMD of 0.29 (0.07, 0.51) (*P* = 0.01) between PCa and non-cancerous tissues for the D^*^ value.

### Detection of Prostate Lesions Using the f Value

**Figure 7:**
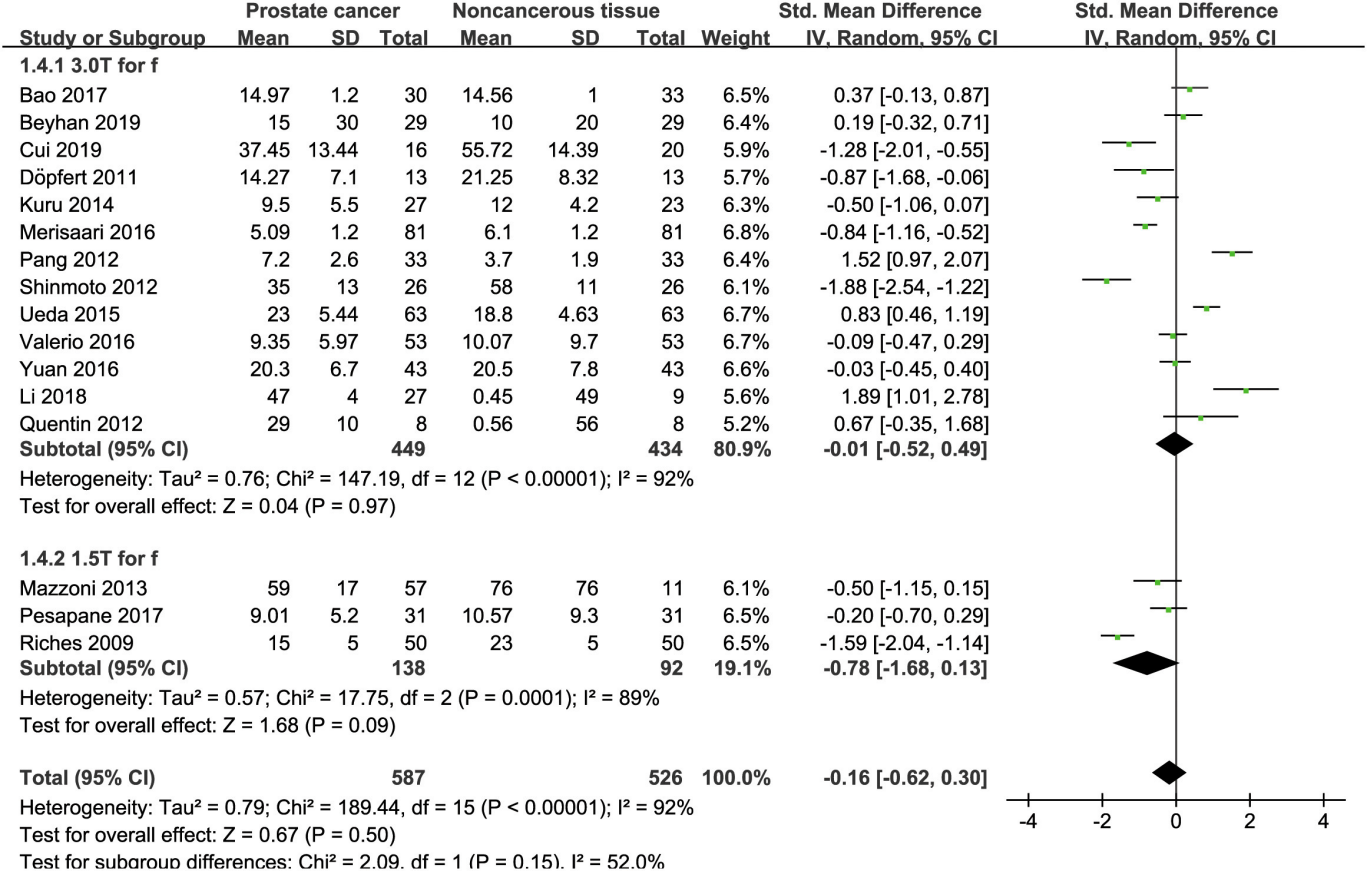
Forest plot of the mean value of the perfusion fraction (f) between prostate cancer and non-cancerous tissues.

Sixteen studies regarding the f value used to detect PCa were included for analysis. The forest plot in Figure 7 shows the distribution of the f value between PCa and non-cancerous tissues. A random-effects model generated an SMD of −0.16 (−0.62, 0.30) (*P* = 0.50) between PCa and non-cancerous tissues for the f value.

### Subgroup Analysis

Three studies are using 1.5 T and 17 studies using 3.0 T for imaging. We merged the results based on different magnetic fields and compared the difference between the two subgroups for ADC, D, D^*^, and f values. From Figures 4–7, the results suggest there is no subgroup difference between 1.5 and 3.0 T in ADC, D, and f values (*P* = 0.14, 0.40, and 0.15, respectively). However, a significant difference is observed in D^*^ value (*P* = 0.02), and the SMD of D^*^ value between PCa and benign tissues at 1.5-T magnetic fields is significantly larger than that at 3.0 T.

### Differentiation Between Low- and High-Grade Prostate Cancer

The identification of tumor aggressiveness is helpful for PCa risk stratification and management. Thus, we further pooled the SMD between low- and high-grade PCa. The results from eight studies suggested low-grade tumors had higher ADC (SMD = 0.63; *P* < 0.001; *I*^2^ = 50%) and D values (SMD = 0.80; *P* < 0.001; *I*^2^ = 52%) than high-grade tumors, but no significant difference was observed in the D^*^ (SMD = −0.22; *P* = 0.16; *I*^2^ = 36%) and f values (SMD = −0.02; *P* = 0.88; *I*^2^ = 10%) (2, 4, 6, 7, 9, 15, 25, 26).

### Diagnostic Performance

**Figure 8:**
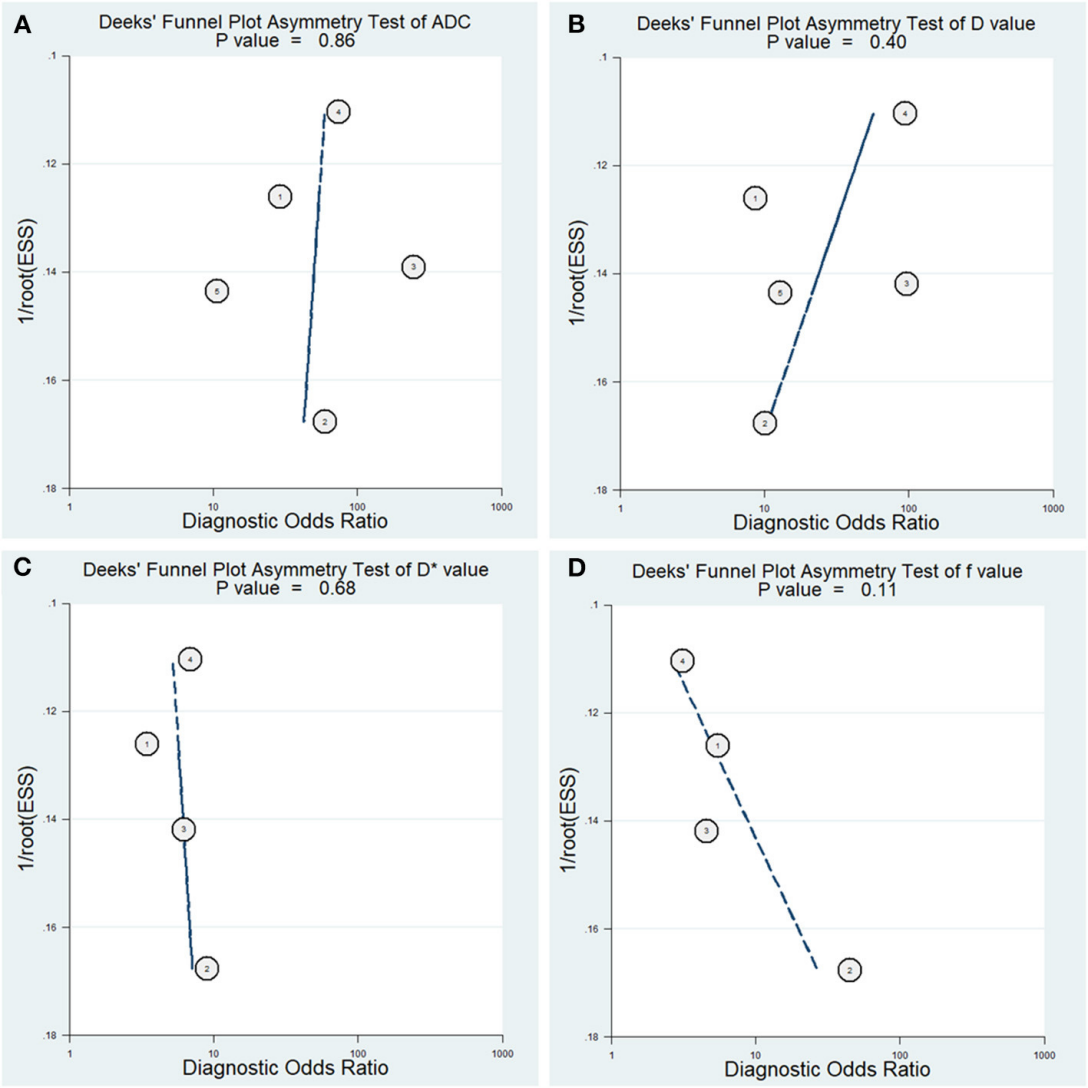
Deeks' funnel plots regarding the diagnostic performance for the **(A)** apparent diffusion coefficient (ADC), **(B)** tissue diffusivity (D), **(C)** pseudodiffusivity (D*), and **(D)** perfusion fraction (f).

**Figure 9:**
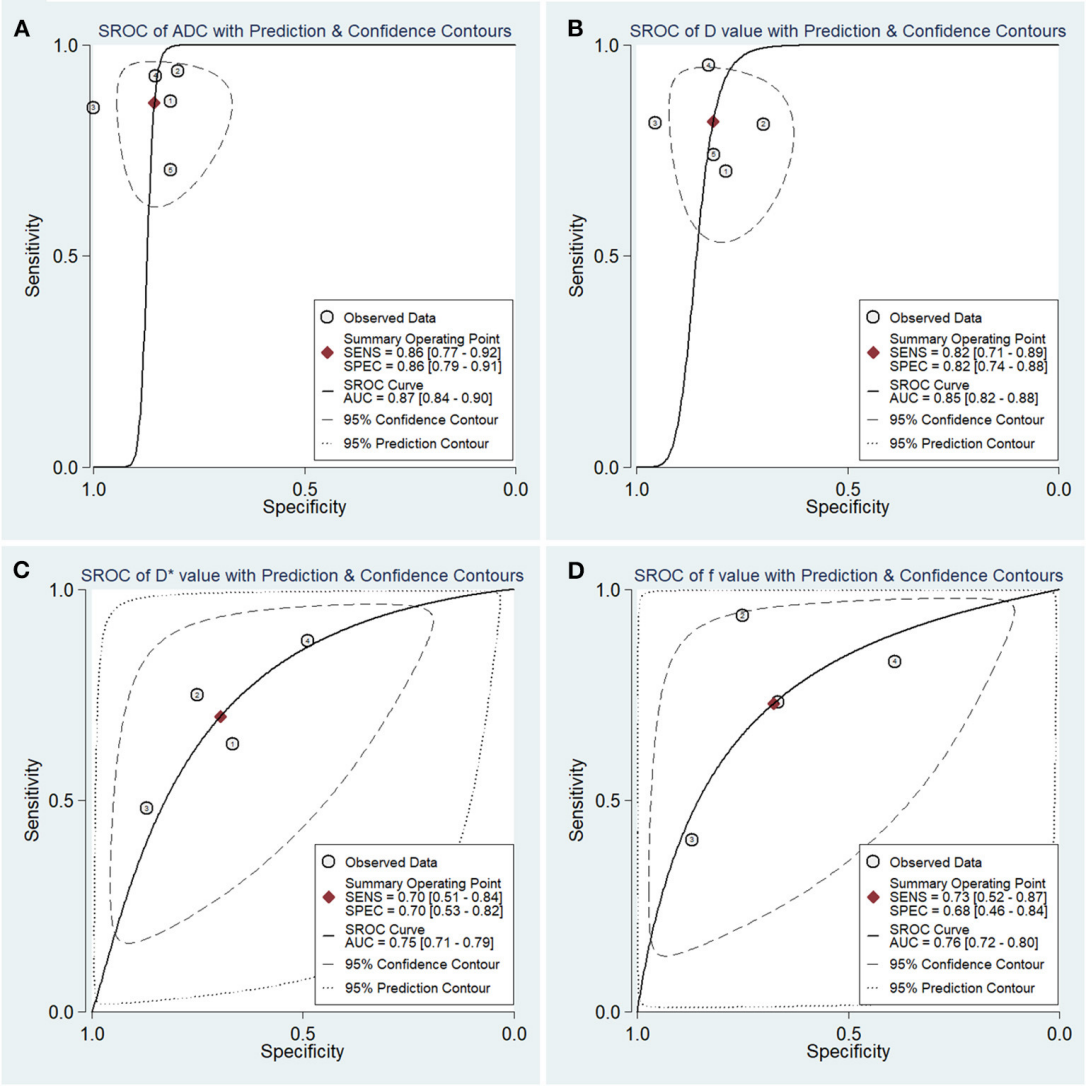
Summary receiver operating characteristic (SROC) curve of the **(A)** apparent diffusion coefficient (ADC), **(B)** tissue diffusivity **(D)**, **(C)** pseudodiffusivity (D*), and **(D)** perfusion fraction (f) in the diagnosis of prostate lesions.

The diagnostic performance with pooled sensitivity, specificity, PLR, NLR, DOR, and AUC of ADC, D, D^*^, and f values are listed in Table 3. Deeks' funnel plots indicated no obvious publication bias in ADC, D, D^*^, and f values (*P* = 0.86, 0.40, 0.68, and 0.11, respectively) (Figure 8). Figure 9 plots the summary receiver operating characteristic curves of ADC, D, D^*^, and f values. Because not all the studies reported the diagnostic performance of IVIM-DWI in the detection of PCa, there were a small number of studies included for analysis in Figures 8, 9. ADC showed comparable diagnostic performance (sensitivity = 86%; specificity = 86%; AUC = 0.87) with the D value (sensitivity = 82%; specificity = 82%; AUC = 0.85), followed by the D^*^ (sensitivity = 70%; specificity = 70%; AUC = 0.75) and f values (sensitivity = 73%; specificity = 68%; AUC = 0.76).

**Table 3:** Pooled estimates and heterogeneity measures for ADC, D, D^*^, and f values.

**Index**	**Sensitivity**	**Specificity**	**PLR**	**NLR**	**DOR**	**AUC**	***I***^****2****^ **(%)**	
							**Sensitivity**	**Specificity**
ADC	0.86 (0.77, 0.92)	0.86 (0.79, 0.91)	6.0 (3.9, 9.1)	0.16 (0.09, 0.27)	37 (17, 82)	0.87 (0.84, 0.90)	48.59%	20.63%
D	0.82 (0.71, 0.89)	0.82 (0.74, 0.88)	4.5 (3.1, 6.7)	0.22 (0.13, 0.37)	20 (9, 46)	0.85 (0.82, 0.88)	53.80%	21.99%
D*	0.70 (0.51, 0.84)	0.70 (0.53, 0.82)	2.3 (1.6, 3.4)	0.43 (0.28, 0.67)	5 (3, 10)	0.75 (0.71, 0.79)	77.27%	71.74%
f	0.73 (0.52, 0.87)	0.68 (0.46, 0.84)	2.3 (1.4, 3.7)	0.40 (0.24, 0.67)	6 (3, 12)	0.76 (0.72, 0.80)	84.85%	82.61%

ADC, apparent diffusion coefficient; D, tissue diffusivity; D^*^, pseudodiffusivity; f, perfusion fraction; PLR, positive likelihood ratio; NLR, negative likelihood ratio; DOR, diagnostic odds ratio; AUC, area under the curve; I^2^, inconsistency index.

### Post-test Probabilities

**Figure 10:**
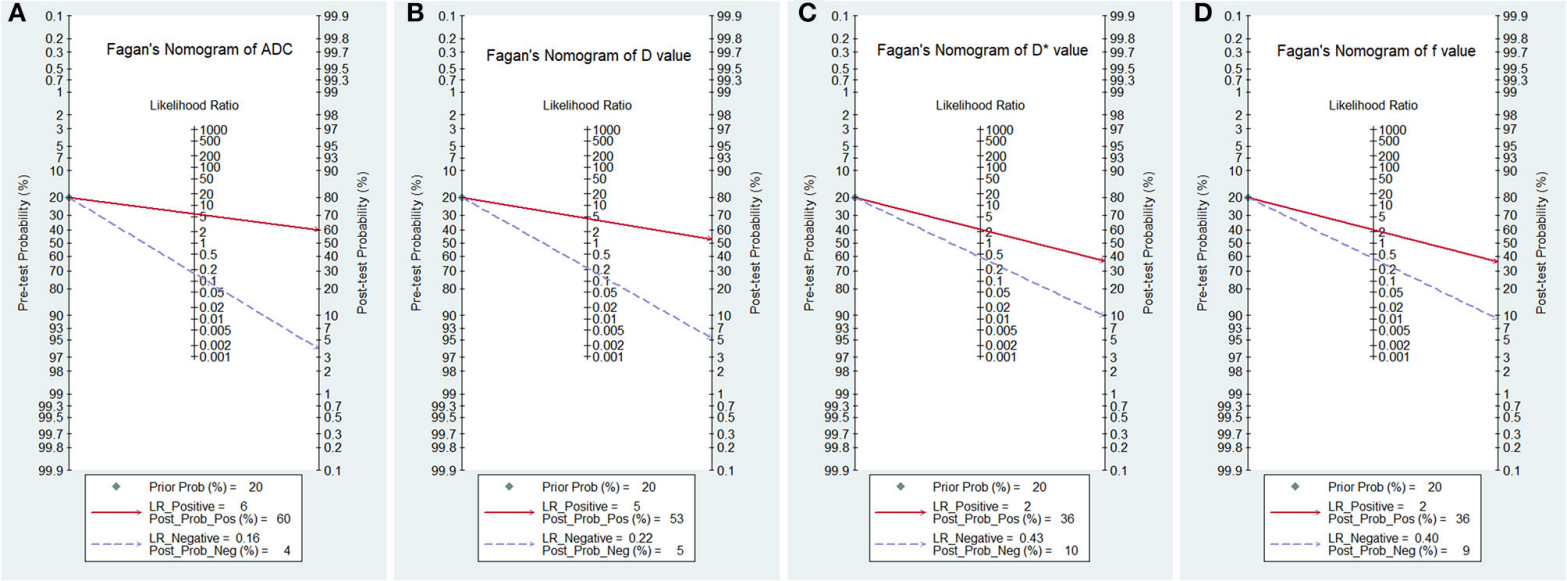
Fagan's nomogram of the **(A)** apparent diffusion coefficient (ADC), **(B)** tissue diffusivity (D), **(C)** pseudodiffusivity (D*), and **(D)** perfusion fraction (f).

Figure 10 is a plot of Fagan's nomograms of ADC, D, D^*^, and f values to predict post-test probabilities. All the pre-test probabilities were set at 20% by default. We regarded the diagnosis of PCa as a positive event, corresponding to lower ADC and D values and higher D^*^ values. Similarly, non-cancerous tissues with higher ADC and D values and lower D^*^ values were regarded as an adverse event. The post-test probability increased to 60% from a pre-test probability of 20% with a PLR of 6.0 and decreased to 4% with an NLR of 0.16, with the prompt of ADC. Thus, the diagnostic preference for PCa will be enhanced using the ADC (a lower ADC) compared with the condition without the prompt of ADC, whose diagnostic probability was set at 20% beforehand. Conversely, the probability of diagnosing PCa will significantly drop from 20 to 4% when an adverse event occurs (a higher ADC). Similarly, the post-test probability of diagnosing PCa will reach 53% with a PLR of 4.5 and drop to 5% with an NLR of 0.22 using D for guidance. The post-test probability of diagnosing PCa will reach 36% with a PLR of 2.3 and drop to 10% with an NLR of 0.43 with the help of D^*^. These data indicated that both ADC and IVIM parameters helped to enhance the accuracy of detecting PCa.

## Discussion

IVIM-DWI is a non-invasive technique that shows superiority in reflecting tumor cellularity and perfusion without requiring a contrast agent. It has already been applied in the differentiation of lung nodules (29), thyroid nodules (30), and breast (31) and brain tumors (32) with good diagnostic performance. To our best knowledge, no prostate study with a large sample size has been reported to determine the value of IVIM to quantitatively distinguish PCa from non-cancerous tissues and identify the tumor Gleason grades, in the background of IVIM becoming a research hotspot in whole-body tumors. Our study provided a timely summary of this issue through pooling all published evidence with strict inclusion criteria and quality assessment. The results showed that the IVIM model has good diagnostic performance but was not superior to the monoexponential ADC value overall.

In this meta-analysis, the SMDs suggested lower ADC and D values in PCa compared with non-cancerous tissues. PCa usually shows an increased cell density and nucleoplasm ratio with active proliferative capacity, which may reduce the extracellular space and restrict the movement of water molecules, causing a reduction in the diffusion coefficient. However, Chatterjee et al. (33) correlated ADC and PCa Gleason grade with three gland component volumes and found that the volumes of the epithelium, stroma, and lumen space had stronger correlations with Gleason patterns and ADC (except stroma volume) than cellularity metrics. These findings indicated the decrease in ADC with increasing PCa Gleason grade can also be attributed to an increased volume of low diffusivity epithelial cells and decreased volumes of higher-diffusivity stroma and lumen space in PCa tissues. The pooled results also suggest excellent diagnostic performance with high sensitivity, specificity, and AUC and increased post-test probability in both ADC and D values, followed by the D^*^ value. Besides, ADC value manifested potential publication bias with *P* = 0.029 of Begg's Test. Publication bias is closely correlated with the high variability of small sample studies and high probability for publishing positive results. As the stronger diagnostic performance of ADC value, it can effectively differentiate PCa with more positive results published in most journals compared with other metrics. Therefore, ADC value may be found potential publication bias in this situation. Including more studies with large sample sizes or negative results that have not been published may solve this problem. A monoexponential model cannot provide an independent perfusion-related parameter and may miscalculate water molecule movement due to a mix with microcirculation perfusion, resulting in an overestimated ADC value (31). Although D value can precisely calculate the true diffusion without the influence of perfusion-related effects, they did not demonstrate superior diagnostic performance compared with ADC. This may largely result from the difference in the number and extent of *b*-values used in the two models. Theoretically, a segmented method is used to fit the IVIM model, expressed as SI/SI_0_ = (1–f) **·** exp(–bD) + f **·** exp(–bD^*^). First, as *b*-value <200 mm^2^/s is referred to low b-value where mainly reflects the pseudodiffusion, and the data in this range are fitted to the bi-exponential model for acquiring D^*^ and f values. Then, the data of b-value higher than 200 mm^2^/s are used to calculate D value using a monoexponential model as the pseudodiffusion from blood flow is negligible in this range (34). Therefore, the monoexponential model is expressed as SI/SI_0_ = exp(–bD) and generates D or ADC value. A larger number and higher *b*-values applied in the IVIM model will significantly prolong the scanning times and introduce motion and susceptibility artifacts, decreasing the sensitivity and accuracy to detect the prostate lesions.

Interestingly, PCa demonstrated a significantly higher D^*^ value but an insignificant f value compared with non-cancerous tissues. Angiogenesis plays a vital role in the growth, progression, and metastasis of PCa (35). A previous meta-analysis indicated that PCa has higher Ktrans, Kep, and Ve than non-cancerous tissue in the peripheral zone using dynamic contrast-enhanced MRI (DCE-MRI) (36). Higher perfusion parameters are expected because of active vasculogenesis and angiogenesis (11), accounting for the increased D^*^ value in PCa. In the subgroup analysis, the SMD of D^*^ value between PCa and benign tissues at 1.5-T magnetic fields is significantly larger than that at 3.0 T, indicating magnetic fields may influence the measurement of D^*^ value. We should be cautious that the number of 1.5-T studies pooled was still not enough to draw a stable result. We observed high variability in the f value, as evident in the large SDs, and both higher and lower mean values of PCa reported among the included studies compared with non-cancerous tissues. Kuru et al. (4) indicated that the f value only reflects the water fraction flowing through the pseudorandomly oriented microvasculature and does not directly correlate with any of the DCE parameters. The f value is also influenced by the transversal relaxation time of the compartments and other bulky flow phenomena (16). Andreou et al. (37) and Liu et al. (31) reported the poor measurement reproducibility of perfusion-sensitive IVIM parameters in liver and breast cancers, likely resulting from the substantially increased heterogeneity of cancers compared with that of normal tissue. Cui et al. (14) investigated whole lesions using histogram analysis and found significant differences between PCa and non-cancerous tissues in the histogram mean, min, 10, 25, 50, 75, 90th, maximum, and skewness of f values, indicating histogram analysis may be a promising method to further excavate the value of perfusion parameters in the prostate.

Our study suggest that both ADC and D values can further discriminate low- from high-grade tumors. Yang et al. (6) reported a D value with excellent diagnostic performance for this discrimination, with 96% sensitivity and 82.9% specificity, suggesting tumor cellularity is correlated with tumor aggressiveness (38). Gibbs et al. (39) found that the mean cell density increased from 14.5% for PCa with a Gleason score 6–21.9% with a Gleason score equal to or larger than eight. Previous studies also indicated that diffusion coefficients correlate with the aggressiveness of PCa (6, 40). However, no significant difference was observed in the f and D^*^ values. This finding suggests that the low diffusion coefficients observed in PCa with high Gleason scores mainly result from pure molecular diffusion rather than perfusion-related diffusion. Furthermore, Kuru et al. (4) demonstrated that perfusion-related parameters could not differentiate between low- and high-grade PCa, whereas Oto et al. (38) also found that quantitative DCE-MRI parameters show no significant correlation with the Gleason scores. These studies further confirmed the results of this meta-analysis.

The ADC, D, D^*^, and f values all demonstrated obvious heterogeneity, which should be further explored. First, the PCa patients had various ages, degrees of prostate-specific antigens, and Gleason scores, which may indicate different tumor aggressiveness and introduce heterogeneity in our meta-analysis. Second, both 1.5-T and 3.0-T MR scanners with various repetition times, echo times, and combinations of *b*-values were used to perform IVIM-DWI in these studies, which may influence accurate calculations of the diffusion and perfusion coefficients and decrease the diagnostic performance compared with monoexponential ADC. Third, the post-processing software and delineations of the regions of interest were different because some studies (7, 14, 23) performed histogram analyses for whole lesions, whereas others delineated the lesions at the largest section as the region of interest. Finally, both benign prostatic hyperplasia and normal tissues in the peripheral zone were treated as non-cancerous tissues, which may encompass different biological characteristics and affect the IVIM values.

The meta-analysis possessed several limitations. First, not all of the studies reported the diagnostic performance of IVIM-DWI to detect PCa. Second, we did not perform a direct comparison with DCE-MRI, which is the gold standard of perfusion imaging and commonly used in the diagnosis of PCa. The issue about whether IVIM-DWI added values to multiparametric MRI in a large sample size remains unclear. Third, the acquisition and analysis of DWI data had not been standardized before pooling, which may influence the wide application of the results.

## Conclusions

IVIM parameters are adequate to differentiate PCa from non-cancerous tissues with good diagnostic performance based on their cellularity and perfusion characteristics, but they are not superior to the ADC value. One unique superiority in the IVIM model is the D^*^ value, which can reflect the perfusion difference between PCa and non-cancerous tissues. Diffusion coefficients rather than perfusion-sensitive parameters can further predict the tumor grades in PCa, which may help risk stratification and clinical management. Histogram analysis may be a promising method to further excavate the value of perfusion parameters in the prostate, but more studies should be included in the future.

## Data Availability Statement

All datasets presented in this study are included in the article/supplementary material.

## Author Contributions

JL and HH conceived the study and revised the manuscript. NH and ZL drafted the manuscript. XL and WD searched the databases and acquired the data. CP and WD performed data analysis and interpretation. All authors contributed substantially to the preparation of the manuscript.

## Conflict of Interest

The authors declare that the research was conducted in the absence of any commercial or financial relationships that could be construed as a potential conflict of interest.

## Abbreviations

AUC: area under the curve
ADC: apparent diffusion coefficient
D: tissue diffusivity
D^*^: pseudodiffusivity
f: perfusion fraction
IVIM-DWI: intravoxel incoherent motion diffusion-weighted imaging
MRI: magnetic resonance imaging
NLR: negative likelihood ratio
SMD: standardized mean difference
I^2^: inconsistency index
PCa: prostate cancer
PLR: positive likelihood ratio.
